# Intrusion Detection Method for Internet of Vehicles Based on Parallel Analysis of Spatio-Temporal Features

**DOI:** 10.3390/s23094399

**Published:** 2023-04-30

**Authors:** Ling Xing, Kun Wang, Honghai Wu, Huahong Ma, Xiaohui Zhang

**Affiliations:** School of Information Engineering, Henan University of Science and Technology, Luoyang 471000, China; xingling_my@163.com (L.X.); wkhaust@163.com (K.W.); mhh@haust.edu.cn (H.M.); zhangxh_1129@126.com (X.Z.)

**Keywords:** Internet of Vehicles, intrusion detection, spatio-temporal features, network security

## Abstract

The problems with network security that the Internet of Vehicles (IoV) faces are becoming more noticeable as it continues to evolve. Deep learning-based intrusion detection techniques can assist the IoV in preventing network threats. However, previous methods usually employ a single deep learning model to extract temporal or spatial features, or extract spatial features first and then temporal features in a serial manner. These methods usually have the problem of insufficient extraction of spatio-temporal features of the IoV, which affects the performance of intrusion detection and leads to a high false-positive rate. To solve the above problems, this paper proposes an intrusion detection method for IoV based on parallel analysis of spatio-temporal features (PA-STF). First, we built an optimal subset of features based on feature correlations of IoV traffic. Then, we used the temporal convolutional network (TCN) and long short-term memory (LSTM) to extract spatio-temporal features in the IoV traffic in a parallel manner. Finally, we fused the spatio-temporal features extracted in parallel based on the self-attention mechanism and used a multilayer perceptron to detect attacks in the Internet of Vehicles. The experimental results show that the PA-STF method reduces the false-positive rate by 1.95% and 1.57% on the NSL-KDD and UNSW-NB15 datasets, respectively, with the accuracy and F1 score also being superior.

## 1. Introduction

The development of the Internet of Things (IoT) and artificial intelligence (AI) technologies has resulted in a gradual increase in the intelligence and networking capabilities of automobiles. As a result, the IoT-based vehicle-to-everything (V2X) communication systems, also known as the Internet of Vehicles (IoV), have become a prominent focus of both academia and industry [1,2,3]. Owing to the advanced wireless communication network technology, vehicles may acquire real-time traffic information, which can improve traffic efficiency. Additionally, through the comprehensive network connections with the outside world, vehicles can elevate their intelligence level further and subsequently provide users with diverse services [4].

With the rapid growth in the number of vehicles in the IoV and the increasingly complex network environment, the emergence of network attacks has introduced considerable security challenges to the IoV, and its data security and communication security are facing significant threats. Once a vehicle is hacked, it will endanger the safety and reliability of the vehicle, resulting in traffic accidents or casualties. For example, hackers can remotely control the vehicle’s brakes, accelerator, steering, engine, and other systems to interfere with or destroy the normal driving of the vehicle. In addition, hackers can also violate the user’s privacy or interests by stealing or tampering with the vehicle’s data. The intrusion detection method can detect whether there is a sign of network attack in the IoV, collect communication flow data in the IoV, and process and analyze these data to realize the classification of intrusion and normal samples. Deploying the intrusion detection method on the vehicle or roadside unit can analyze and judge the communication data incoming to the vehicle or roadside unit. If abnormal or attack behavior data are found, it can refuse to receive or forward the data, thereby detecting attacks in the IoV.

The IoV intrusion detection methods can mainly be divided into methods based on traditional machine learning and methods based on deep learning. Methods based on traditional machine learning manually extract features using decision trees (DT)s, support vector machines (SVM), etc., and classify normal sample data and attack sample data. These methods have certain defects (low detection performance and long processing time) when dealing with massive and multidimensional intrusion detection data of the IoV. At present, deep learning is the primary means to realizing the intrusion detection of the IoV. Mining the intrusion sample data through deep learning can obtain hidden features, thereby detecting network intrusion data. Several deep learning models, including convolutional neural network (CNN) [5], autoencoder (AE) [6], recurrent neural network (RNN) [7], long short-term Memory network (LSTM) [8], generative adversarial network (GAN) [9], and deep belief network (DBN) [10], are used to implement IoV intrusion detection methods. These schemes have achieved good intrusion detection performance. However, the high false-positive rate has consistently been the main problem these deep learning-based intrusion detection schemes have faced for the IoV. This is because these solutions do not fully extract the data features in the IoV; moreover, the correlation between features has yet to be fully considered. In response to the above problems, some researchers have proposed to combine CNN and LSTM [11] to extract the spatio-temporal behavior features in the IoV data to improve the intrusion detection performance and reduce the false-positive rate. However, these schemes usually concatenate the two models, ignoring the differences and respective advantages between temporal and spatial features. Additionally, the features extracted by the previous model will affect the latter model. When the previous model has limitations, the intrusion detection performance of the entire model will be affected.

In order to address the challenges IoV intrusion detection faces, we propose a IoV intrusion detection method based on parallel analysis of spatio-temporal features (PA-STF). Firstly, We propose a correlation-based feature selection method to select features that are highly correlated with behavior categories and construct the optimal feature set, thereby reducing the feature dimension. Then, we use Temporal Convolutional Network(TCN) and LSTM to extract spatial and temporal features in a parallel manner. Finally, we fuse the extracted spatio-temporal features based on the self-attention mechanism and input them into a multi-layer perceptron(MLP) for intrusion detection. The main contributions of this paper are as follows:

- We propose a correlation-based feature selection method for selecting relevant features suitable for intrusion detection in the IoV. A recursive elimination method is used to obtain the optimal feature set, thereby effectively eliminating redundant features.
- We design a new spatio-temporal feature parallel extraction architecture, using TCN and LSTM to extract spatio-temporal features of IoV traffic in parallel. The proposed architecture has higher reliability compared with the serial architecture.
- We design a spatio-temporal feature fusion approach using the self-attention mechanism. By giving attention weights to the spatio-temporal features, it is possible to fuse various features efficiently. The fusion features significantly enhance the IoV intrusion detection model’s efficacy.
- We conduct experimental evaluation on the intrusion detection dataset. Compared with the comparison methods, our method has higher accuracy and F1 score, and has lower false-positive rate.

## 2. Related Work

Deep learning can effectively learn the inherent laws of sample data. It can adapt to higher-dimensional learning and prediction requirements by constructing a nonlinear network structure composed of multiple hidden layers. Some researchers [12,13,14] analyze the traffic and speed of vehicles in the Internet of Vehicles through deep learning methods and edge computing technologies, so as to provide drivers with personalized safety information, which provides a data basis for intrusion detection in the Internet of Vehicles. These studies [15,16,17] all mentioned that the deep learning method could improve the detection performance of intrusion detection, so it is widely used in intrusion detection of the IoV. Yang et al. [18] proposed an intrusion detection method for in-vehicle networks based on federated deep learning, using the periodicity of network messages, using the ConvLSTM model to detect network intrusions, and training the intrusion detection model based on federated deep learning. Li et al. [19] proposed an intrusion detection scheme for the IoV based on transfer learning, using two modes of cloud-assisted update and local update. Shone et al. [20] proposed an unsupervised deep learning intrusion detection technology using an asymmetric deep autoencoder to build a classification model. However, this method needs better classification performance in unbalanced samples. Xu et al. [21] designed a Log-Cosh variational autoencoder method, which uses a logarithmic hyperbolic chordal function to design a loss term to generate diverse intrusion data, thereby improving detection accuracy. However, these deep learning-based solutions still have a high false-positive rate, caused by insufficient extraction of relevant features of the IoV.

The intrusion data in the IoV contains many spatio-temporal features, which can reflect some attacker characteristics. Therefore, this prompts researchers to use deep learning methods such as CNN or LSTM to extract and process these spatio-temporal features. Hu et al. [22] built a technique for detecting intrusions. Use CNN with a split convolution module to improve the variety of spatial characteristics and decrease the influence of information redundancy across channels on the model. Park et al. [23] converted the network traffic into a grayscale image, established a Siamese CNN based on the small sample learning method, and determined the attack type according to the similarity score of the attack samples. In order to capture the time-dependent dynamic features in network traffic, Zhou et al. [24] proposed an incremental LSTM network intrusion detection method, which introduces state changes into LSTM and processes network data by obtaining the hidden layer state of LSTM dynamic information. Ashraf et al. [25] combined LSTM and autoencoder to extract the timing features in the network traffic of the IoV, which improved the accuracy of intrusion detection in the IoV. The above schemes usually only use CNN or LSTM to process spatio-temporal features, and there is a problem of insufficient feature extraction. For this reason, some researchers use both CNN and LSTM to build a hybrid model to extract spatio-temporal features. Wang et al. [26] proposed a hierarchical intrusion detection system based on spatio-temporal features. Firstly, CNN is used to learn the spatial features in network traffic packets, and then LSTM is used to learn the temporal features between multiple network traffic packets. Finally, a more accurate spatio-temporal feature vector is obtained. Nonetheless, the aforementioned solutions disregard the problem of variable time intervals between data packets in the flow. To address this issue, Han et al. [27] suggested a spatially and temporally aware intrusion detection model. They developed a time- and length-sensitive LSTM method for acquiring a wider range of temporal characteristics from intermittent streams.

The intrusion detection method based on deep learning realizes the efficient detection of network attacks in the IoV. However, these artificial intelligence-based methods also bring many risks and challenges, such as adversarial sample attacks and the security of the intrusion detection system itself. Achieving more reliable artificial intelligence schemes through formal methods [28,29] has attracted extensive attention from researchers. By using mathematical logic, models, and proofs to check whether the IoV intrusion detection system satisfies the design specification, errors in the system can be found, thereby improving the security and reliability of intrusion detection.

In general, current intrusion detection methods based on spatio-temporal features usually use deep learning methods such as CNN and LSTM to establish serial intrusion detection models. However, the overall performance of these methods is easily affected by the previous model and ignores the spatio-temporal features. The spatial-temporal features extracted need to be more comprehensive. Therefore, we extract spatio-temporal features in a parallel manner, avoiding the negative impact of a single model in serial feature extraction on the whole and giving full play to the advantages of spatio-temporal features. Thus, the IoV’s effectiveness in detecting intrusions is enhanced, and the number of false positives is reduced.

## 3. Proposed PA-STF Method

**Figure 1:**
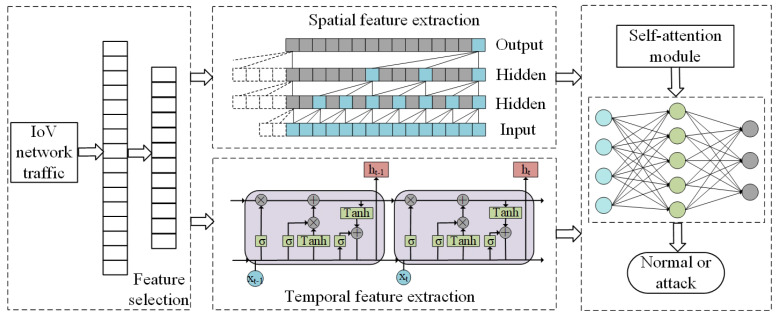
Architecture of the PA-STF method.

Figure 1 shows the overall architecture of our proposed method. The PA-STF method is mainly divided into three parts. In the first part, we preprocess the IoV traffic, obtain the network traffic’s original characteristics, and select features based on the correlation method to obtain the best feature subset. In the second part, TCN and LSTM are used concurrently to extract spatio-temporal characteristics from the preprocessed data. In the third part, we employ the self-attention mechanism to determine the relative relevance of spatio-temporal information, combine them, and use them as the input to the multilayer perceptron to judge whether the detected traffic is normal or attacked. Each part is described in detail in the next section.

### 3.1. Data Preprocessing

Since deep learning models cannot directly process character features [30], we first use label encoding to convert character features in the dataset into numerical features that deep learning models can process. As significant numerical differences between features will harm the model, we need to normalize the data to limit the feature values within a specific range so that the model can converge more quickly [31]. We use the min-max method to achieve normalization which is calculated as follows:

(1) $$x^{\prime }=\frac{x-\min(x)}{\max(x)-\min(x)}$$

where *x* is the input vector; max(x) and min(x) are the maximum and minimum values of the input, respectively; and $x^{\prime }$ is the normalized data.

Due to the complex and multidimensional nature of the data in the IoV, direct use of these data for model training consumes excessive computing resources, and redundant features are likely to cause model overfitting [32]. Hence, we present a correlation-based technique for reducing feature dimension, thereby improving the intrusion detection ability of the model. The correlation-based feature selection method obtains the feature set with the highest correlation with intrusion detection. When the feature has a high correlation with the class label, we can consider the feature to be more suitable for predicting the class label.

Assume that each instance $x_{i}=\left\{f_{1}^{i},f_{2}^{i},...,f_{k}^{i}\right\}$ in the data set $X=\left\{x_{1},x_{2},...,x_{n}\right\}$ has *k*-dimensional features, where *f* are the features, *i* is the *i*-th instance of the dataset, and (1≤i≤n), *n* is the number of instances in the dataset. For an instance $x_{i}$, we use ${\mathrm{cor}}_{m,n}^{i}=\left(f_{m}^{i},f_{n}^{i}\right)$ to represent the correlation between any two features. From this, we can obtain the feature correlation matrix *C*, which is expressed as follows:

(2) $$C=\left[\begin{array}{cccc} {\mathrm{cor}}_{1,1}^{1} & {\mathrm{cor}}_{1,2}^{1} & \cdots & {\mathrm{cor}}_{1,k}^{1}\\ {\mathrm{cor}}_{2,1}^{2} & {\mathrm{cor}}_{2,2}^{2} & \cdots & {\mathrm{cor}}_{2,k}^{2}\\ \vdots & \vdots & \ddots & \vdots \\ {\mathrm{cor}}_{k,1}^{n} & {\mathrm{cor}}_{k,2}^{n} & \cdots & {\mathrm{cor}}_{k,k}^{n} \end{array}\right]$$

We use the Pearson correlation coefficient to calculate the correlation between the features and class label, and the calculation method is as follows:

(3) $${\mathrm{cov}}_{j,k}^{i}=\frac{1}{n}\sum \left(f_{j}^{i}-\mu_{j}\right)\left(f_{k}^{i}-\mu_{k}\right)$$

(4) $${\mathrm{cor}}_{j,k}^{i}=\frac{{\mathrm{cov}}_{j,k}^{i}}{\sigma_{j}*\sigma_{k}}$$

Among them, $f_{j}^{i}$ and $f_{k}^{i}$ are feature *j* and feature *k* that need to calculate correlation, *n* is the total number of samples, μ is the mean value, and σ is the standard deviation. After calculating the correlation between each feature value and class label in the feature correlation matrix, we need to sort them to find the best feature subset. For this, we use a recursive feature elimination method to remove the least relevant features in each iteration and finally obtain the top most relevant features. The correlation-based feature selection algorithm is shown in Algorithm 1.
**Algorithm 1** Correlation-Based Feature Selection Algorithm**Input:** *T*: training data set; *K*: original feature set; *D*: feature subset;**Output:** best feature subset $A_{d}$; 1:Use the feature set *K* to train on the training data set *T*;2:Calculate the correlation of each feature with the class label;3:**for** d∈D **do**4:    **while** k∈K **do**5:        Sort feature sets based on correlation;6:        Get the last feature *k* of the original feature set;7:        $D\left(i\right)\leftarrow k_{l}$;8:        K[m−k+1]←D(i);9:        $D\left(A_{d}\right)\leftarrow D\left(A_{d}\right)-D\left(i\right)$;10:    **end while**11:**end for**12:Return the best subset of features $A_{d}$

### 3.2. Parallel Extraction of Spatio-Temporal Features

In the communication traffic of the Internet of Vehicles, we usually extract the features of intrusion data based on the basic features, content features, and statistical features of the network traffic. Among these features, the same type of features will show a few aggregation rules, thus presenting certain spatial features. TCN has a better effect on local spatial feature extraction through the receptive field, so TCN can be used to process and extract the spatial features in the traffic of the Internet of Vehicles; Moreover, the Internet of Vehicles traffic is a kind of time series data, and the time dependence in the data can provide useful support for intrusion detection. LSTM is very suitable for processing time data and extracting temporal features from it. Extracting spatio-temporal features can discover deep-level Internet of Vehicles traffic behavior mode, which is of great significance to the intrusion detection of the Internet of Vehicles.

(1) TCN-based spatial feature extraction: Traditional spatial feature extraction methods usually use the CNN model, a computational model that imitates the structure of biological neural networks and consists of convolutional layers, pooling layers, and fully connected layers. By superimposing the number of network layers, the fitting performance of the model to the data can be enhanced, and so CNN has achieved good results in speech recognition and image recognition. However, too many network layers will make the model parameters overly numerous and easily cause the model to overfit. The extended model training time is unsuitable for the IoV environment. As a new structure of CNN, temporal convolutional network (TCN) enables the model to process sequence data by adding causal convolution, and the proposed expanded convolution and residual modules enable it to have the ability to memorize historical information. Compared with the traditional CNN, which needs to process network traffic data into two-dimensional images for spatial feature extraction, TCN can directly extract spatial features from one-dimensional data, which has the advantages of fewer computing resources and stable gradients. Therefore, our proposed method uses TCN to extract the characteristics in the IoV traffic. The specific process is as follows.

The TCN model uses a one-dimensional fully convolutional network to extract spatial features from the data. The causal convolution structure is used in the TCN model, and the sequence data can be modeled by making the output of the current layer only depend on the convolution of the previous layer and the previous t time. The following formula can express the causal convolution operation of TCN:

(5) $$F*X\left(x_{t}\right)=\sum_{n=0}^{N}f_{n}\cdot x_{t+n-N}$$

where F represents the filter set, F$=\left\{f_{1},f_{2},...,f_{N}\right\}$, *f* is the individual filter, and *N* is the number of filters; X is the input sequence, X$=\left\{x_{1},x_{2},...,x_{T}\right\}$, where *x* is the input item, and *T* is the size of the input sequence; and ∗ is the convolution operation, and *n* is the size of the convolution kernel. When the scale of input data continues to expand, the number of convolutional network layers also increases, which give rise to a number of problems such as complex training and gradient disappearance. To this end, the TCN model introduces the concept of dilated convolution, which stipulates that the convolution input must be sampled at intervals so that the effective window size increases exponentially with the increase in the number of network layers; thus, only a few convolution layers are needed to obtain a larger receptive field. The expansion convolution operation on the *t*-th element in the input sequence can be expressed by the following formula:

(6) $$F\left(t\right)=\sum_{i=0}^{k-1}f\left(i\right)\cdot x_{t-d\cdot i}$$

Among these, *k* is the size of the filter, *i* is the current filter size, $x_{t}$ is the *t*-th element of the input sequence, *f* is the convolution operation, *d* is the defined expansion factor used to control the sampling rate when d=1 is a conventional convolution operation, and t−d·i represents the past direction.

The residual connection in TCN is used to improve the generalization ability of CNN so that more spatial feature information can be retained in extracting spatial features of IoV traffic, thereby improving the performance of the intrusion detection model. The following formula can express the residual module:

(7) res=Act(X+f(X))

where X is the input sequence of the model, f(X) is the output after dilated convolution, and Act is the activation function. The activation function often employs the ReLU function, which has the benefits of a fast computation speed and convergence speed of gradient descent, but there is also a dead ReLU issue, in which the gradient is always 0 throughout the backpropagation process when the input is negative. In order to solve the above problems, we propose to use the Leaky ReLU activation function to replace the ReLU activation function in the original TCN to solve the gradient disappearance problem. Leaky ReLU can be expressed by the following formula:

(8) $$\mathrm{LeakyReLU}\left(x\right)=\left\{\begin{array}{c} x\\ ax \end{array}\;\;\right.\begin{array}{c} x>0\\ x\leq 0 \end{array}=max(0,x)+\gamma min(0,x)$$

where γ is a hyperparameter and is set to 0.1, *x* is the input data, and *a* is the slope of the activation function when the input is negative. When the input is negative, Leaky ReLU will provide a smaller linear value, so that a positive gradient is obtained, thereby alleviating the zero gradient problem caused by negative input.

The IoV traffic data are input into the TCN module, and the spatial feature vector of the IoV data can be extracted. Compared with traditional CNN, TCN has a flexible receptive field and stable gradient, which can enhance the feature extraction ability. The extracted features can train a higher-performance intrusion detection model.

**Figure 2:**
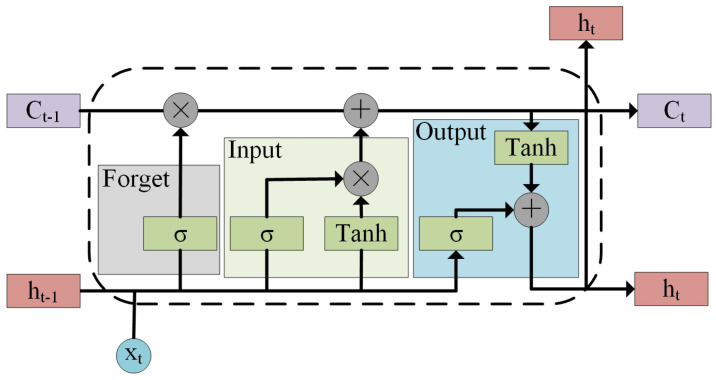
LSTM unit structure.

(2) LSTM-based temporal feature extraction: Temporal feature extraction usually uses RNN because RNN can process sequence data with changing characteristics, in contrast to general neural networks. As an improved version of RNN, LSTM can solve the long-term dependency problem in long-sequence training [33] and has been widely used by researchers. Therefore, we use LSTM to extract the temporal features in the IoV data. The LSTM model structure is composed of units, and the LSTM unit is shown in Figure 2. Each unit mainly includes three parts: forget gate, input gate, and output gate. The control of the state of the LSTM unit is realized through these three gates.

The forget gate is mainly used to control whether the unit discards the unit state of the previous layer, which the following formula can express:

(9) $$f_{t}=\sigma \left(W_{f}\cdot \left[h_{t-1},x_{t}\right]+b_{f}\right)$$

where σ is the sigmoid activation function, *t* is the current moment, *f* is the forget gate, $x_{t}$ is an element in the input sequence, $h_{t-1}$ is the state of the preceding sequence, $W_{f}$ is the weight, and $b_{f}$ is the bias. The forget gate outputs a vector ranging from 0 to 1, which is used to control the information of the units in the previous layer: 0 is no reservation, while 1 is all reservations.

The input gate is used to process the current sequence input, which can be expressed by the following formula:

(10) $$i_{t}=\sigma \left(W_{i}\cdot \left[h_{t-1},x_{t}\right]+b_{i}\right)$$

(11) $$a_{t}=tanh(W_{C}\cdot \left[h_{t-1},x_{t}\right]+b_{C})$$

Among these, $i_{t}$ is the input gate, and σ is the sigmoid activation function. It can be seen that the input gate is divided into two steps: the first step uses the Sigmoid activation function, and the second step uses the tanh activation function to obtain the candidate unit information $a_{t}$.

The final output will be affected by the forget gate and the input gate. The output gate processing process is expressed by the following formula:

(12) $$C_{t}=C_{t-1}*f_{t}+i_{t}*a_{t}$$

(13) $$o_{t}=\sigma \left(W_{o}\left[h_{t-1},x_{t}\right]+b_{o}\right)$$

(14) $$h_{t}=o_{t}*tanh(C_{t})$$

Here, $C_{t}$ is the current unit information; $C_{t-1}$ is the previous unit information; $W_{o}$ and $b_{o}$ are weight and bias, respectively; and ∗ is the Hadamard product.

When the IoV data are input into the LSTM model, the transmission status of the data can be controlled according to its internal gate to realize long-term memory and forget unimportant information. The LSTM model can effectively extract the temporal features of sequence data. These features are input into the subsequent part of our proposed method, described in the next section, for fusion to build an intrusion detection model with higher detection performance.

### 3.3. Spatio-Temporal Feature Fusion and Intrusion Detection

After we use TCN and LSTM to extract the spatio-temporal features in the Internet of Vehicles data in parallel, we need to input them into MLP for intrusion detection model training. However, due to the different emphases of spatio-temporal features, they are not all equally important for distinguishing different attack types. of. For example, the three features of synack, ackdat, and tcprtt in the traffic of the Internet of Vehicles all reflect the time-dependent characteristic state of the TCP connection, which has a strong correlation for detecting time-sensitive attacks. The detection performance of this type of attack can be improved if the weight of its related features is increased. Therefore, we adopt the self-attention mechanism to distinguish the importance of different features. We compress different components into a single representation by assigning attention weights to spatio-temporal features and dynamically adjust the features according to the semantic and scale information of different spatio-temporal feature weights to achieve spatio-temporal feature fusion. The self-attention mechanism makes the fused spatio-temporal features suppress useless information, thus having a stronger representation ability for normal and attack traffic.

First, we need to input the extracted spatio-temporal features into the self-attention module to calculate the attention score of the spatio-temporal features. The spatio-temporal feature *F* is input to the attention module for linear mapping to obtain the query matrix *Q* and the key-value pair matrices *K* and *V*. Assuming that there is a query $q\in R^{q}$ and key-value pairs $\left(k_{1},v_{1}\right),\ldots ,\left(k_{n},v_{n}\right)$, where $k_{i}\in R^{k}$ and $v_{i}\in R^{v}$, the attention score is calculated as follows:

(15) $$attention\left(q,\left(k_{1},v_{1},\ldots ,k_{n},v_{n}\right)\right)=\sum_{i=1}^{n}\mathrm{softmax}\left(\frac{q_{,}k_{i}}{\sqrt{d_{k}}}\right)v_{i}$$

Through the softmax operation of the inner product of *q* and each $k_{i}$, the similarity between *q* and each $v_{i}$ is obtained, where $\sqrt{d_{k}}$ is a scaling factor to prevent the gradient of the softmax function from disappearing, *n* is the number of key-value pairs, $k_{i}$ is the *i*-th key, and $v_{i}$ is the *i*-th value. Through the self-attention module, we can assign attention scores to spatio-temporal features and fuse the features extracted with the TCN and LSTM models according to the attention scores. The feature fusion method can be expressed by the following formula:

(16) $$F_{fusion}=\lambda_{TCN}F_{spatial}+\lambda_{LSTM}F_{temporal}$$

where $F_{fusion}$ is the fused feature; $F_{spatial}$ and $F_{temporal}$ are the spatial features and temporal features extracted by the TCN model and the LSTM model, respectively; and $\lambda_{TCN}$ and $\lambda_{LSTM}$ are their respective weights. Fusion weights are normalized using the softmax method. The feature fusion method based on the self-attention mechanism adds weight constraints to the extracted spatio-temporal features of the IoV communication traffic. This method can obtain information more conducive to intrusion detection and classification.

Based on the features obtained by the above spatio-temporal feature fusion process, we input them into a multilayer perceptron neural network to complete the classification task of intrusion detection in the IoV. We use the cross-entropy loss function as the loss function of the multilayer perceptron. The fusion of spatio-temporal features makes the model complex and can easily cause over-fitting. Therefore, we add an L2 regularization term to the loss function to reduce over-fitting. The degree of integration reduces the complexity of the model. The loss function is defined as follows:

(17) $$L=\frac{1}{N}\sum_{i}L_{i}+\eta {∥\omega ∥}^{2}=-\frac{1}{N}\sum_{i}\sum_{c=1}^{M}y_{ic}\log\left(p_{ic}\right)+\eta {∥\omega ∥}^{2}$$

Here, *M* is the number of categories, $y_{ic}$ is a sign function, *y* is the predicted probability distribution, *p* is the real probability distribution, *i* is the index of the sample, *c* is the index of the category, and *N* is the total number of samples. When the real category of sample *i* is equal to *c*, it takes 1; otherwise, it takes 0. Furthermore, $p_{ic}$ is the predicted probability that sample *i* belongs to category *c*, ω is the model parameter, and η is the penalty item. Through continuous training iterations, the loss function of the model is converged so as to obtain the optimal intrusion detection model.

## 4. Performance Evaluation

### 4.1. Dataset and Evaluation Metrics

We selected the NSL-KDD dataset [34] and UNSW-NB15 dataset [35] to test the intrusion detection performance of our proposed method. The NSL-KDD dataset is one of the most commonly used datasets in the field of network intrusion detection. In the NSL-KDD dataset, each piece of network traffic data consists of 42-dimensional features, of which 38 dimensions are digital features, 3 dimensions are character features, and the remaining dimension is the label feature. The first 41-dimensional features can be divided into basic, content feature, and traffic features. The basic feature contains the basic information of each traffic data, and the content feature is mainly the payload information of the data packet, which can be used to represent the network behavior information. The traffic characteristics are some traffic statistics, such as time-based or host-based traffic statistics. The attack behaviors in the dataset are mainly divided into four types: DoS, Probe, U2R, and R2L.The specific attack types and descriptions of the data set are shown in Table 1. The UNSW-NB15 dataset contains three types of basic features, content features, and flow features. Specifically, the five flow identities include srcip, destip, sport, dsport, and proto, the 13 basic features include state, sbyte, dbyte, and sttl, among others; and the eight content features include swin, dwin, and stcpd, among others. The traffic features include nine time-related features and the remaining additional features. The specific sample category descriptions in the dataset are shown in Table 2.

The proposed method was implemented using Python language, the Numpy library to preprocess the data set, and Scikit-learn and Tensorflow to realize the spatio-temporal feature extraction and intrusion detection classification model based on deep learning. The main parameter settings of the model are shown in Table 3.

We used accuracy, FPR, and F1 score to evaluate the performance of the proposed intrusion detection method. Among these, accuracy is the proportion of the correctly classified samples to all samples, FPR is negative samples predicted as the proportion of positive samples to the total negative samples, and F1 score is the harmonic mean of precision and recall. The calculation method is as follows:

(18) $$Accuracy=\frac{TP+TN}{TP+FN+FP+FN}$$

(19) $$FPR=\frac{FP}{FP+TN}$$

(20) $$F1=\frac{2TP}{2TP+FP+FN}$$

Here, TP is the true-positive examples, FP is the false-positive examples, TN is the true-negative examples, and FN is the false-negative examples.

### 4.2. Performance Comparison

The methods we used in our comparison were as follows: SVM [36], which uses support vector machines, a traditional machine learning method, to classify packets as trusted or malicious; CNN [37], which uses only convolutional neural network performs spatial feature extraction and uses the loss function and redundant error items designed by the spatial characteristics of the link load to achieve intrusion detection; LSTM [38], which only uses long short-term Memory network training to obtain the characteristics of traffic data changes in the time dimension In order to achieve intrusion detection; and CNN-BiSRU [39], which uses two deep learning models for serial feature extraction, with the first being convolutional neural network to extract the spatial features of the original data and the other being bidirectional simple recurrent unit to extract the temporal features based on it. Finally, the classification results were output through softmax to achieve the purpose of intrusion detection.

Table 4 shows the comparison of the intrusion detection performance of each method. In the NSL-KDD dataset, it can be seen that the Accuracy, F1, score and FPR of the PA-STF method are optimal compared to the other schemes. The PA-STF method achieved an accuracy of 98.68%, which is 3.34% higher than that of the next best method. Meanwhile, the F1 score of the PA-STF method reached 98.94%, which was 2.73% higher than that of the next best method. In the NSL-KDD dataset, the false-positive rate of the PA-STF method was only 0.21%, which remained extremely low and 1.95% lower than that of the next lowest method.

Table 5 shows the specific values of the intrusion detection performance of the above method on the UNSW-NB15 dataset. It can be seen that the accuracy of the PA-STF method was higher than that of the comparison method, reaching 96.34%, and the next highest was 94.22% of that the CNN-BiSRU method. Our method was 2.12% higher than that of the best comparison method. Our method has a solid correct classification ability. PA-STF is also the best performer on the false-positive rate indicator, at only 1.38%. The false-positive rate was reduced by 1.57% compared with the method with the lowest false-positive rate. For the F1 score, our proposed PA-STF method achieved an F1 score of 95.76%, which was 1.52% higher than the state of the art. According to the comparative data, the method based on deep learning can better model the spatio-temporal features of the IoV, and the comprehensive detection performance is stronger. Our method has a high accuracy rate in the intrusion detection of the IoV and can effectively reduce false positives during the detection process with higher usability.

**Figure 3:**
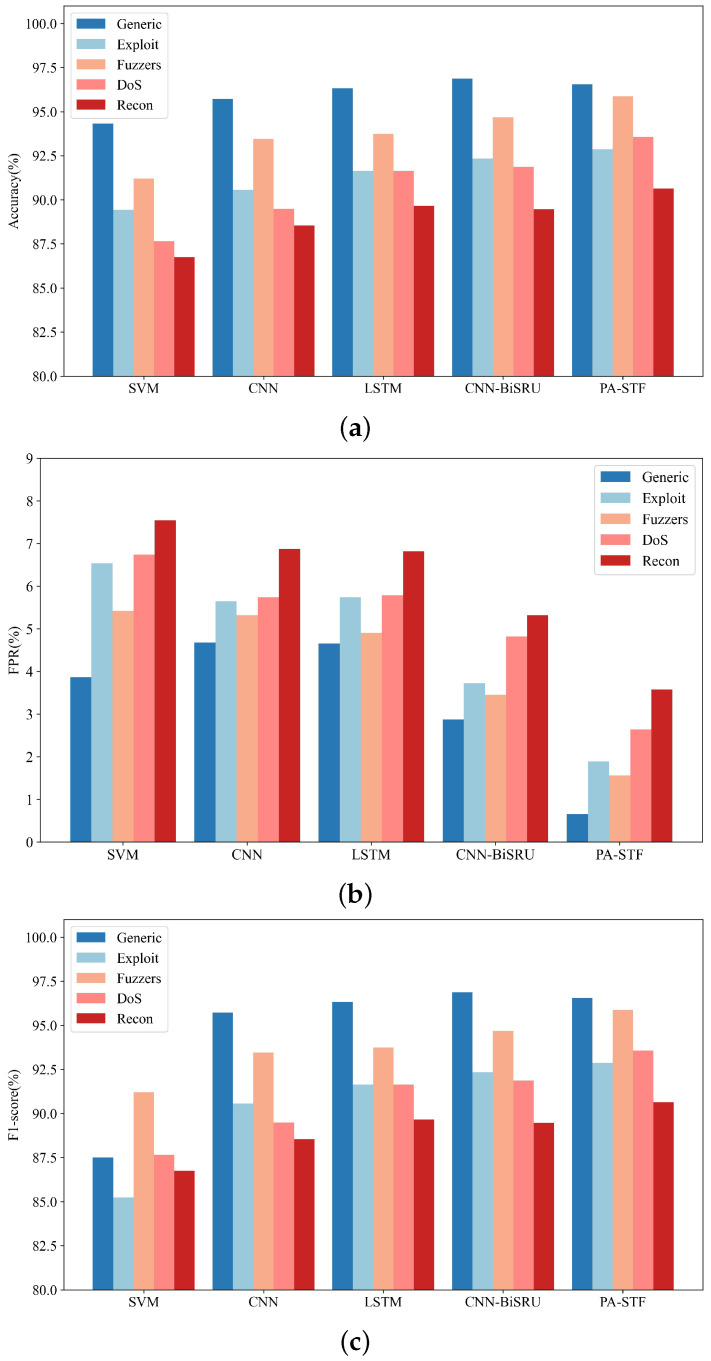
Performance comparison of each method under different attack types: (**a**) comparison of accuracy, (**b**) comparison of FPR, and (**c**) comparison of F1-score.

We compared the detection performance of each method for different attack types. We selected five attack types with the largest sample data in the UNSW-NB15 dataset. Figure 3 compares each method’s intrusion detection performance on different attack types. Figure 3a shows that the proposed PA-STF method maintains the highest accuracy rate in the four types of attack: Exploit, Fuzzers, DoS, and Recon. Although the accuracy rate of the generic attack is not the highest, it is the same as that of the best compared method, CNN-BiSRU. The difference is negligible. We determined that this is because the generic attack is an information collection type attack, and the proposed method produces more false negative results when detecting this type of attack. Figure 3b compares the false-positive rate of each method under different attack types. It can be seen that the false-positive rate is the lowest for the five attack types. The false-positive rate is the lowest among intrusions, and the false-positive rate of the Recon intrusion is the highest among all attacks. Our analysis is related to the number of samples in the data set. When the number of samples of this type of attack is too small, the false-positive rate will increase. The advantages of the PA-STF method can be clearly seen in Figure 3c, in which the F1 scores of the five intrusion types are the highest among all the methods. The PA-STF has a good detection effect on spatio-temporal feature modeling. In contrast, as a traditional machine learning method, SVM has a lower F1 score than do the other deep learning methods. We attributed this to SVM’s inability to fully extract spatio-temporal features.

**Figure 4:**
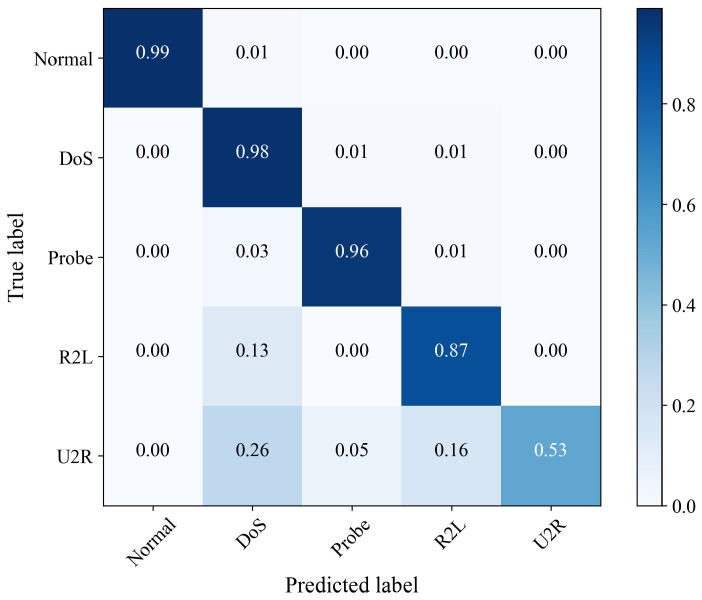
Confusion matrix of the PA-STF method on NSL-KDD dataset.

Figure 4 is the confusion matrix obtained using the proposed method on the NSL-KDD dataset. It can be seen that the proposed method achieved a correct recognition rate of 99% for normal samples in the NSL-KDD dataset. Moreover, 98% of DoS attacks could be detected correctly, and 96% of Probe attacks could be detected correctly. Due to the small sample size of R2L and U2R, the detection rate was low. However, the proposed method had a very low probability of misidentifying the four attack types as normal, making the false-positive rate of the proposed method very low, which proves the effectiveness of the proposed method.

**Figure 5:**
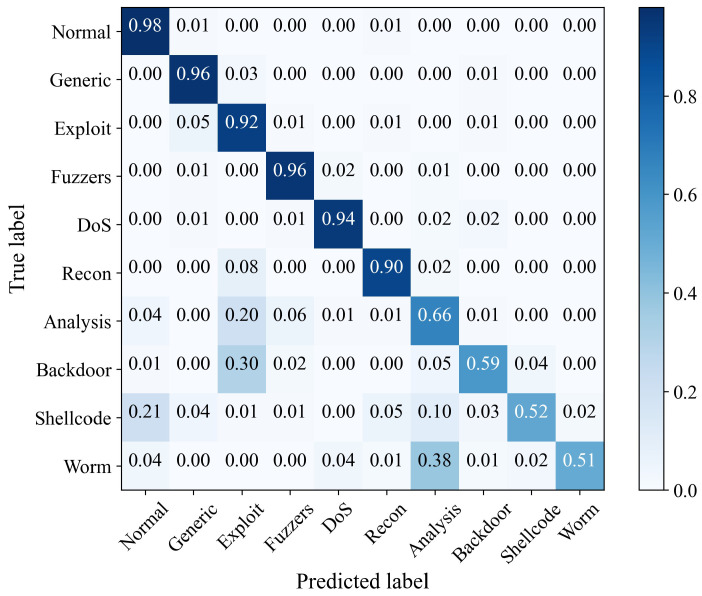
Confusion matrix of the PA-STF method on UNSW-NB15 dataset.

Figure 5 is the confusion matrix of the PA-STF method on the UNSW-NB15 dataset for classifying various types of attacks. The ordinate of the confusion matrix is the true sample data label, and the abscissa is the predicted sample data label. In each square, the darker the color is, the more samples of this type are classified. According to the confusion matrix, we can intuitively see the intrusion detection of various label types in the UNSW-NB15 dataset. We performed normalization processing in the row direction of the confusion matrix. It can be seen that 98% of the normal sample data were correctly classified. Moreover, the PA-STF model had a high correct detection rate for several attack types with a large amount of data. For attacks with small sample sizes, the detection rate was low. The PA-STF method still has certain limitations for small sample data. When the sample data of the attack type is too small, this type of behavior’s representation and generalization ability could be better because the model does not fully extract its spatio-temporal features. For Shellcode attacks, the PA-STF model has specific false positives, and some Shellcode attacks will be identified as normal samples. However, the overall PA-STF method still has strong intrusion detection performance.

**Figure 6:**
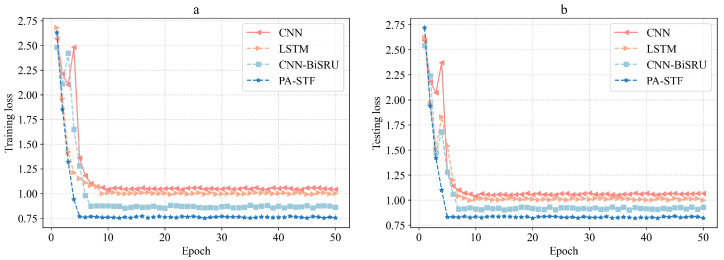
Training and testing losses of the different methods: (**a**) training loss comparison and (**b**) test loss comparison.

Figure 6 shows the convergence of the loss functions of several deep learning methods. It can be seen that the PA-STF method requires only a small number of training epochs, the training loss can converge to about 0.75, and the other methods require more training epochs to achieve convergence. Furthermore, PA-STF exhibits smaller training and testing losses compared to CNN, LSTM, and CNN-BiSRU. The experiments shows that the method proposed in this paper can better fit the data because PA-STF performs feature selection based on feature correlation, which reduces the dimension of input data.

**Figure 7:**
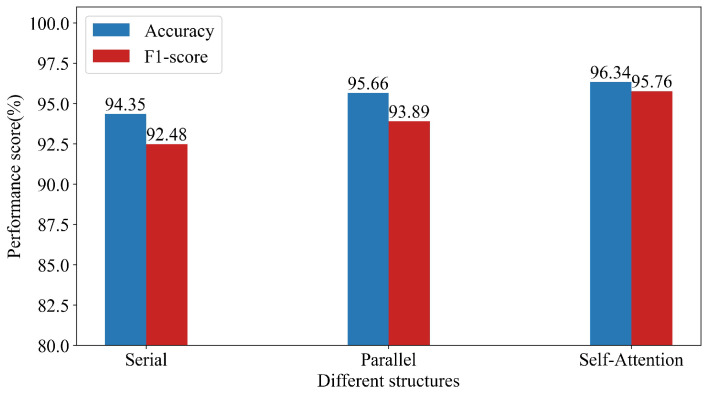
Performance comparison of the different structures.

Figure 7 shows the performance of the different structures, where Serial represents the use of TCN for spatial feature extraction first and then LSTM for temporal feature extraction. Parallel represents the use of TCN and LSTM for parallel spatio-temporal feature extraction. Self-Attention represents the use of TCN and LSTM to extract spatio-temporal features in parallel and then the use of the self-attention mechanism for feature fusion. Parallel feature extraction has certain advantages in intrusion detection performance compared with serial feature extraction. After the addition of the self-attention mechanism for feature fusion, our method improved by 1.99% and 3.28% in accuracy and F1 score, respectively, compared with serial feature extraction, which reflects the effectiveness of the PA-STF method based on the self-attention mechanism for spatio-temporal feature fusion.

**Figure 8:**
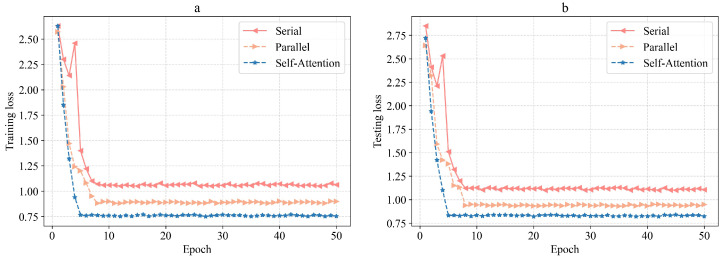
Training and testing losses of different structures: (**a**) training loss comparison and (**b**) test loss comparison.

Figure 8 shows the convergence of the loss functions of different structures. It can be seen that the loss of the parallel structure based on self-attention is significantly lower than that of the serial and no self-attention structure and has a faster convergence speed, which shows that it can better fit the data. The feature fusion structure based on parallel the spatio-temporal feature extraction and self-attention mechanism proposed in this paper can obtain spatio-temporal feature information more effectively, avoiding the problems of the serial extraction structure being affected by the previous submodel, so this method has better performance.

We compared and evaluated the applicability of the proposed method to the IoV in terms of the memory footprint and the time required for intrusion detection. Table 6 shows the comparison of the memory footprint of each method and the average detection time per message. It can be clearly seen from the table that the memory usage and detection time of the proposed method were lower than those of the serial feature extraction method CNN-BiSRU. According to our analysis, this is because the complexity of the TCN model in the proposed method is lower than that of CNN, and the parallel feature extraction method can use multithreaded parallel computing to further improve the model computing efficiency. Therefore, the proposed method takes less memory and requires less detection time. This shows the superiority of our parallel feature extraction. Although the proposed method has higher memory usage than does the CNN method and LSTM method that only use a single deep learning model (this is due to the complexity of the proposed method model itself being higher than that of a single deep learning model), but the detection time is lower. Moreover, the intrusion detection performance of the proposed method is better than that of the method using only a single deep learning model, so the proposed method is more suitable for the IoV environment that is sensitive to detection performance and detection time. Although the memory footprint and detection time of the SVM method are much lower than those of the proposed method, the intrusion detection performance of the SVM method is lower for the network data presenting complex and multidimensional characteristics in the IoV environment, which is not applicable in the real IoV environment.

In general, our method requires less memory and less detection time while obtaining excellent intrusion detection performance, which reflects the effectiveness of the proposed method. How to further reduce the average detection time to improve the response speed of intrusion detection in the IoV is a problem worth exploring. Reducing memory usage to achieve a lightweight intrusion detection model requires further research. In the proposed method, we propose a correlation feature selection method to construct the optimal feature subset to reduce the dimension of the input data, which is beneficial to decreasing memory usage and detection time. Additionally, model compression and parameter quantization can further optimize intrusion detection speed and memory usage.

## 5. Conclusions

In this paper, we propose an IoV intrusion detection method based on the parallel analysis of spatio-temporal features. Our method extracts spatio-temporal features from complex multidimensional IoV traffic by using TCN and LSTM in a parallel manner. Compared with previous methods that use a single deep learning model to extract features or serial feature extraction methods, our method extracts spatio-temporal features more fully, thus effectively improving the intrusion detection performance of the IoV and reducing the occurrence of false positives. First, we perform feature selection based on the feature correlation of IoV traffic to obtain the best feature subset. Then, unlike previous methods that use a single model or serially extract spatio-temporal features using CNN or LSTM, we use TCN and LSTM to extract spatio-temporal features in the IoV traffic in parallel. Finally, we use the self-attention mechanism to fuse the extracted spatio-temporal features and input them into a multilayer perceptron network for intrusion detection. We conducted experiments on the NSL-KDD and UNSW-NB15 datasets, and the experimental results show that our method can achieve better intrusion detection results in less time than can previous methods, which validates our method’s effectiveness.

Although our method has achieved good results in detection performance and detection speed, with the rapid increase of the number of vehicles in the IoV and the increasingly complex network environment, higher requirements are being placed on the response speed of intrusion detection. Edge intelligence can achieve high bandwidth and low latency for data transmission by migrating data and artificial intelligence to the edge of the network [40,41]. Therefore, in future research work, we will consider introducing edge intelligence into IoV intrusion detection to improve its processing speed. How to introduce other types of features to further improve the performance and robustness of intrusion detection is also worthy of further research. We will consider using new feature extraction techniques to incorporate features such as behavior and context into the IoV intrusion detection model together with existing spatio-temporal features so as to realize a more effective IoV intrusion detection model.

## Figures and Tables

**Table 1:** Data types and sizes of the NSL-KDD dataset.

Attack Type	Size	Description
Normal	77,054	Normal behavior data
DoS	53,385	Denial of service attacks
Probe	14,077	Probing and scanning attacks
R2L	3649	System vulnerability attacks
U2R	252	Gain access to remote

**Table 2:** Data types and sizes of the UNSW-NB15 dataset.

Attack Type	Size	Description
Normal	2,218,761	Normal behavior data
Generic	215,481	Information gathering attacks
Exploit	44,525	Attacks that exploit known system vulnerabilities
Fuzzers	24,246	Fuzzy attacks
DoS	16,353	Denial-of-service attacks
Recon	13,987	Port scanning attacks
Analysis	2677	HTML file penetration attack
Backdoor	2329	Attacks that bypass to systems
Shellcode	1511	Exploit code attacks
Worm	174	Self-replicating and spreading-malware attacks

**Table 3:** Main parameters of the model.

Parameter	Value	Description
Learning-rate	0.01	Gradient descent steps during model training
Epoch	50	Number of training rounds
Dropout	0.2	Dropout rate of neural network unit
TCN-layer	4	Number of layers of the TCN model
LSTM-Unit	48	Number of LSTM model units
MLP-layer	4	Number of layers of the MLP model

**Table 4:** Performance comparison of the different methods on the NSL-KDD dataset.

Methods	Accuracy	FPR	F1 Score
SVM	89.64	5.64	87.63
CNN	93.17	4.38	92.69
LSTM	92.77	5.34	92.83
CNN-BiSRU	95.34	2.16	96.21
PA-STF	98.68	0.21	98.94

**Table 5:** Performance comparison of different methods on the UNSW-NB15 dataset.

Methods	Accuracy	FPR	F1 Score
SVM	92.64	4.65	86.75
CNN	94.09	4.68	90.24
LSTM	93.77	4.71	92.45
CNN-BiSRU	94.22	2.95	94.24
PA-STF	96.34	1.38	95.76

**Table 6:** Comparison of the memory footprint and detection time of each method.

Methods	Memory Footprint (KB)	Detection Time (ms)
SVM	352	0.23
CNN	2194	1.79
LSTM	4627	2.14
CNN-BiSRU	8463	5.31
PA-STF	5216	1.45

## Data Availability

The data used to support the findings of this study can be downloaded from https://research.unsw.edu.au/projects/unsw-nb15-dataset, accessed on 21 May 2022.

## Abbreviations

The following abbreviations are used in this manuscript:

IoV	Internet of Vehicles
IoT	Internet of Things
AI	artificial intelligence
V2X	vehicle-to-everything
DT	decision tree
SVM	support vector machine
TCN	temporal convolutional network
RNN	recurrent neural network
LSTM	long short-term memory
CNN	convolutional neural network
AE	autoencoder
GAN	generative adversarial network
DBN	deep belief network
MLP	multilayer perceptron
BiSRU	bidirectional simple recurrent unit
PA-STF	parallel analysis of spatio-temporal features
FPR	false-positive rate
